# This baby is not for turning: Women’s experiences of attempted external cephalic version

**DOI:** 10.1186/s12884-016-1038-1

**Published:** 2016-08-26

**Authors:** N. P. Watts, K. Petrovska, A. Bisits, C. Catling, C. S. E. Homer

**Affiliations:** 1 Centre for Midwifery, Child and Family Health, Faculty of Health, University of Technology Sydney, Sydney, Australia; 2 Royal Hospital for Women, Sydney, Australia

**Keywords:** External cephalic version, Breech presentation, Pregnancy, Qualitative research, Caesarean section

## Abstract

**Background:**

Existing studies regarding women’s experiences surrounding an External Cephalic Version (ECV) report on women who have a persistent breech post ECV and give birth by caesarean section, or on women who had successful ECVs and plan for a vaginal birth. There is a paucity of understanding about the experience of women who attempt an ECV then plan a vaginal breech birth when their baby remains breech. The aim of this study was to examine women’s experience of an ECV which resulted in a persistent breech presentation.

**Methods:**

A qualitative descriptive exploratory design was undertaken. In-depth semi-structured interviews were conducted and analysed thematically.

**Results:**

Twenty two (*n* = 22) women who attempted an ECV and subsequently planned a vaginal breech birth participated. Twelve women had a vaginal breech birth (55 %) and 10 (45 %) gave birth by caesarean section. In relation to the ECV, there were five main themes identified: ‘seeking an alternative’, ‘needing information’, ‘recounting the ECV experience’, ‘reacting to the unsuccessful ECV’ and, ‘reflecting on the value of an ECV’.

**Conclusions:**

ECV should form part of a range of options provided to women, rather than a default procedure for management of the term breech. For motivated women who fit the safe criteria for vaginal breech birth, not being subjected to a painful experience (ECV) may be optimal. Women should be supported to access services that support vaginal breech birth if this is their choice, and continuity of care should be standard practice.

## Background

The optimal mode of birth for women who have a baby in the breech position at term is controversial. Since the Term Breech Trial [1], the availability of planned vaginal breech birth has diminished [2]. In Australia, in 2012, 87 % babies in the breech position were born by caesarean section (CS) [3]. In an attempt to reduce the need for CS for breech, external cephalic version (ECV) has become a popular, safe practice and is recommended for women who have a straightforward pregnancy with a breech presentation near term [4–8]. The procedure is offered to women from around 36 weeks gestation with success at early or late gestation being equivocal [9]. Success rates for ECVs are reported between 40 and 60 % [10]. As such, an ECV has been identified as a potential way to reduce CS rates [11] although consideration of the subsequent care for women whose babies remain breech is important.

There are few studies about a woman’s experience surrounding an ECV and her subsequent preference for mode of birth [12–14]. These report on the experiences of women who have a persistent breech post ECV and then gave birth by CS, or on women who had successful ECVs that resulted in plans for a vaginal birth. There is a paucity of understanding about the experience of women who attempt an ECV then choose to plan a vaginal breech birth when their baby remains breech. Therefore, the aim of this study was to examine women’s experience of an ECV that resulted in a persistent breech presentation. This is part of a wider program of research exploring the experiences of women who choose a vaginal breech birth and the midwives and doctors who cared for them [15–17].

## Methods

A qualitative descriptive study was undertaken [18, 19]. This approach was selected to enable an accounting of events from the participants of the study in order to better understand their experiences. The participants were the women who had experienced an ECV and while their stories are described and explored, the findings seek to interpret meanings and actions from those stories.

This study received approval from the Human Research Ethics Committee-Northern sector, South Eastern Sydney Local Health District, New South Wales Health. Reference: HREC 12/072 (HREC/12/POWH/163). Recruitment took place between March and October 2013 from two hospitals in New South Wales that supported planned vaginal breech birth.

English-speaking women, who after an unsuccessful ECV planned a vaginal breech birth for a singleton pregnancy in the past 7 years regardless of their eventual mode of birth, were recruited in 2013. Women were identified from two Australian public maternity units in urban/metropolitan areas that supported women to have a vaginal breech birth. A review of the hospitals’ database that recorded women who planned a vaginal breech birth was undertaken to identify eligible women. In total, 32 women were invited to participate with 22 (69 % response rate) willing to be interviewed.

Two members of the research team conducted all the interviews. These took place in women’s homes as that was identified as the most convenient. Appointments were made with women at a time most suitable to them and usually family members or partners cared for the children while the interview took place. A series of open-ended questions were asked during interview, which lasted around 60 min. Each were recorded with a digital voice recorder and transcribed by a professional transcription service. Data collection ended when no new information arose from the interviews and it was agreed by the research team that data saturation had been achieved.

We used a similar process to that reported in our previous paper [15]. An inductive thematic analysis was used to identify, describe, and analyse themes and patterns within the data [20]. The process meant that transcriptions were initially read and re-read by three members of the research team and codes were identified. The codes were then examined for patterns and the underlying meaning of the issues identified were analysed within and between transcripts. The codes were refined and then grouped according to commonalities which gave themes. These were shared with the research group again and then cross-reviewed with the data, carefully considering counterexamples or negative cases to ensure that the similarity and diversity of experiences were identified [21].

The themes are illustrated with quotes from the data. At the end of each quote is a code indicating the interview number and whether they had a caesarean section (CS) or a vaginal birth (VB).

## Results

**Fig. 1:**
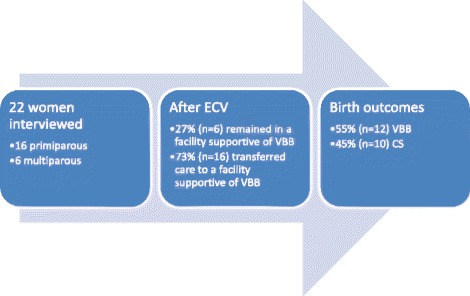
Participants outcomes

Twenty-two women were interviewed, of which three quarters were primiparous (*n* = 16; 73 %). All were Caucasian, and the majority were educated to tertiary level. Most women were interviewed in the 3 years since their breech birth. At the time of the ECV, most (*n* = 16; 73 %) women were attending a hospital that did not support vaginal breech birth. After the breech presentation persisted, these women were informed that they would need a CS and all decided to actively seek different carers to facilitate vaginal breech birth. The other six (27 %) women were receiving care at a hospital that supported vaginal breech birth and continued with this care. Overall outcomes of birth were that twelve (55 %) women achieved a vaginal breech birth and 10 (45 %) gave birth by CS after labour had commenced (see Fig. 1).

Five main themes were identified. These were ‘seeking an alternative’, ‘needing information’, ‘recounting the ECV experience’, ‘reacting to the unsuccessful ECV’ and, ‘reflecting on the value of an ECV’.

### Seeking an alternative

Most women sought out other means to turn the baby and viewed the ECV as their last resort. Most tried more than one alternative therapy prior to attempting an ECV, some of which consisted of acupuncture, chiropractic, hypnotherapy, moxibustion, maternal positioning and yoga. This was motivated by the desire to avoid a medical procedure (ECV) and ultimately to avoid a CS birth. One woman said:

*“So I thought the whole breeching thing - I’ll fix that easy. It’s not a problem. I just do acupuncture, I do chiropractic, I do my exercises, he’s going to turn’. That was my attitude. And I thought he was going to turn all the way up to, the D-Day but…yeah. That didn’t happen.”* CS3

### Needing information

Women needed detailed individualised information to assist their decision-making to have an ECV. The way information was presented by clinicians made a difference to the way women felt about attempting an ECV. For example:

*“When he offered the option he did it in such a way that it was so welcoming, it didn’t feel like it was a procedure and he assured me it would be up to the point that he felt the most pressure that he felt would be safe for the baby. So that was a different way in which the ECV was painted and so I agreed to do the ECV.”* CS15

Despite this positive comment, many other women believed they were given insufficient information by their clinicians about the risks and benefits of an ECV. One woman expressed this by saying:

“*I felt that I wasn’t adequately educated as to what it was going to be like. I think I would have preferred a more complete understanding of the process before I made the choice more than just being told ‘it’s not pleasant’.”* VB10

Many women sought additional information from the internet, social media and their friends and family regarding ECV. Information on the internet was mostly reported as negative. This clouded the perception of the value of attempting an ECV. One woman said:

*“I stupidly Googled it before I went and I find that people only want to share their stories when they are horrific.”* VB11

Some women felt they were not given a choice to opt out of an ECV, but that it was an expected step in the course of management for a breech baby. One woman expressed this lack of choice saying:

*“I don’t think it was really presented as an option it was just presented as the next step.”* VB17

### Recounting the ECV experience

When asked how they felt about the experience of having the ECV performed, the majority of women responded that the procedure caused them physical pain. Because of the subjective nature of the experience of pain, some of the women compared it to other painful events in their life, like childbirth. For example:

*“I can do pain. I didn’t have drugs for my first labour and I didn’t have drugs for my second labour.. but that was, incredibly painful.”* VB1

Some women continued to have pain for some time after the procedure. One woman said:

*“I remember going to bed afterwards and trying to sleep ‘cause I was just in so much pain. And it really felt like someone had pummelled me, I had been through an absolute wringer.”* VB22

### Reacting to an unsuccessful ECV

The reaction to a persistently breech baby was mixed. Most women had a strong emotional response, but reactions varied dependent upon where the ECV was attempted. For women who were already in a hospital supportive of vaginal breech birth, the significance of an unsuccessful ECV was negligible as the option of vaginal birth was already in place prior to the procedure. For the women who were not given this option, disappointment and distress were reported. The availability of vaginal breech birth for these women was crucial to how they felt about the outcome of the ECV. These women expressed feelings of disappointment, devastation and unacceptance of the consequence of having a baby remaining in the breech position. For example:

*“I definitely didn’t accept it. And I remember when I came home from the ECV that had failed and I was, you know, again, wailing and crying. Just absolutely devastated.”* VB17

Women, cared for in hospitals that did not support vaginal breech birth, hinged their hopes on a successful ECV as they felt it was the last resort to be able to continue along their path of planning a vaginal birth. One women woman said:

“*It was awful. I was quite traumatised after that [ECV]. I think also knowing that this was my last chance, if he didn’t turn that I would have to have a caesarean.”* VB14

In comparison, women who were aware of the option of vaginal breech birth prior to ECV were not as concerned when the procedure did not turn the baby. For example:

*“We walked away from that and I think at that point I began to accept that she wasn’t going to turn back. And that I was going to be delivering her breech [vaginally].”* CS5

### Reflecting on the value of ECV

Almost half of the women (46 %) said they would not attempt an ECV in a future pregnancy. Reasons for this were three-fold. Firstly, the experience caused physical pain. This was expressed by one woman as:

*“So they’re pushing your stomach around and it’s just bloody excruciating… it just didn’t feel good… Never, never, never, again will I ever, ever, do that, never.”* VB10

Secondly, ECV was seen as a procedure that introduced excessive risk to the baby that the women considered unnecessary, and some felt guilty for this. For example:

*“felt really guilty that I’d possibly brought a little bit of distress to my baby in utero.. would never attempt an ECV if I was breech second time around.”* VB2

Thirdly, the option and availability of a planned vaginal breech birth meant the women who were already in a hospital supportive of vaginal birth did not appreciate that an ECV to promote cephalic presentation was worthwhile because the fetal presentation was inconsequential for their pregnancy and birth. Many women who sought out the option of vaginal breech birth commented that if the option of vaginal breech birth had have been presented and available prior to attempting an ECV then they may not have chosen to attempt it. One woman said:

*“I think I wish I’d had more information about the ECV and that it’s not necessarily something that you need to do.. so from what I know now, I wouldn’t necessarily make that choice.”* VB8

## Discussion

This study describes Australian women’s experiences who underwent an ECV which resulted in a baby who remained a breech presentation. Other studies have described women’s experiences of having a breech presenting baby [22–24] and ECV [13, 14, 25–27]. Our findings add to the understanding of women’s experiences with a breech-presenting baby in the late third trimester of pregnancy as many women are offered an ECV,

It was common for women to seek out a variety of complementary therapies for the relief of pregnancy-related complaints and symptoms [28], and this included turning babies [23]. This study showed that women used alternative therapies to attempt spontaneous cephalic version. However, few were reported in the literature as having any major effect on turning babies, although moxibustion (a Chinese herbal medical intervention that stimulates acupuncture points) may have some effect in reducing CS rates when used in conjunction with positional therapies [29, 30].

Alternative therapies can also be used to reduce the discomfort of an ECV. Women were not given analgesia during the ECV in this study, although methods to reduce discomfort can include hypnosis [31], inhalation analgesia, [32], injectable analgesia, and regional anaesthesia [33]. However, pain levels vary in women and women’s perception and recollection of pain are important. Vlemmix et al. [25] concluded that a woman’s willingness to agree to undergo an ECV is influenced by her perception of the pain and the likely success of converting her baby to a cephalic presentation. Women’s recollection of pain can also be diminished if their ECV was successful [26]. This may explain why the majority of women in this study reported it as a painful experience, as they all had an unsuccessful ECV, although this memory may have been compounded by the distress three quarters of them felt when they were told to expect a CS.

The way information and options for birth were presented was important. The timing, manner and content of the information were central to women’s levels of anxiety. Discussions with health professionals surrounding myths (predominately found on the internet) and risks and benefits for ECV, vaginal breech birth and caesarean section were appreciated. In particular, risks and benefits are useful when articulated in a clear, easy to understand format especially the success rates. ECV success rates of 40–60 % have been reported [10], however it is pertinent to reveal local rates to women as well as discuss rates for women’s individual breech-positioned baby (eg. frank, footling). For example, footling breech babies have 2.77-times more likelihood of remaining cephalic after ECV than babies in a frank breech position [34]. Information to aid decision-making can also be facilitated through a decision-making tool. For example, a small study in Canada using an audio-guided workbook for women with a breech presentation was found to be helpful, although self-rated anxiety levels were not lowered significantly after using the decision aid [35].

Breech positions have long been seen as ‘malpositions’ of the fetus. It could be argued that there is a place for respecting women’s choice to refuse ECV and try for a vaginal breech birth. Other countries, such as Finland, where one in three women with breech babies are eligible and willing to try for a vaginal birth have this attitude towards breech-positioned babies [36]. Furthermore, another study by the same authors suggested that a trial of vaginal breech birth is as positive as a vertex birth experience for women [22]. Considering this, it may be time to move away from the approach of women being told their babies are in the ‘wrong’ position, and that turning them to a cephalic presentation is the only desirable option.

This study included a selective group of women because they were highly motivated to pursue a vaginal breech birth. This may not reflect the general population of women, many of whom consent to a caesarean section when their baby is persistently breech. Despite this, these women provide a unique opportunity to understand the experience of women who have an ECV that does not turn their baby. Qualitative studies such as this provide a window into the experience for these women and could be reflective of other women’s experiences.

## Conclusion

Understanding women’s experiences is important for doctors and midwives who provide care for women with a baby in the breech presentation late in pregnancy. ECV should form part of a range of options provided to women, rather than a default procedure for management of the term breech. For motivated women who fit the safe criteria for vaginal breech birth, not being subjected to a painful experience (ECV) is optimal. Given many women reported that they would not attempt an ECV in a future pregnancy, access services that support vaginal breech birth needs to be made available.
